# Impact of a Local Low-Cost Ward-Based Response System in a Canadian Tertiary Care Hospital

**DOI:** 10.1155/2016/1518760

**Published:** 2016-10-17

**Authors:** Andrea Blotsky, Louay Mardini, Dev Jayaraman

**Affiliations:** ^1^Department of General Internal Medicine, Montreal General Hospital, McGill University Health Centre, Montreal, QC, Canada; ^2^Department of Critical Care, Montreal General Hospital, McGill University Health Centre, Montreal, QC, Canada; ^3^Department of Critical Care, Royal Victoria Hospital, McGill University Health Centre, Montreal, QC, Canada; ^4^Department of Critical Care, Jewish General Hospital, Montreal, QC, Canada

## Abstract

*Background*. Medical emergency teams (METs) or rapid response teams (RRTs) facilitate early intervention for clinically deteriorating hospitalized patients. In healthcare systems where financial resources and intensivist availability are limited, the establishment of such teams can prove challenging.* Objectives*. A low-cost, ward-based response system was implemented on a medical clinical teaching unit in a Montreal tertiary care hospital. A prospective before/after study was undertaken to examine the system's impact on time to intervention, code blue rates, and ICU transfer rates.* Results*. Ninety-five calls were placed for 82 patients. Median time from patient decompensation to intervention was 5 min (IQR 1–10), compared to 3.4 hours (IQR 0.6–12.4) before system implementation (*p* < 0.001). Total number of ICU admissions from the CTU was reduced from 4.8/1000 patient days (±2.2) before intervention to 3.3/1000 patient days (±1.4) after intervention (IRR: 0.82, *p* = 0.04 (CI 95%: 0.69–0.99)). CTU code blue rates decreased from 2.2/1000 patient days (±1.6) before intervention to 1.2/1000 patient days (±1.3) after intervention (IRR: 0.51, *p* = 0.02 (CI 95%: 0.30–0.89)).* Conclusion*. Our local ward-based response system achieved a significant reduction in the time of patient decompensation to initial intervention, in CTU code blue rates, and in CTU to ICU transfers without necessitating additional usage of financial or human resources.

## 1. Introduction

Medical emergency teams (METs), which are also known as rapid response teams, comprise groups of health care professionals who respond to high acuity cases in an effort to decrease the risk of further patient deterioration. An important principle underlying rapid response systems is that early intervention can improve patient outcomes [1, 2]. Though the MERIT trial, a major multicenter, cluster-randomized controlled trial, failed to demonstrate a benefit of rapid response teams [3], indirect data from the literature surrounding METs suggest that earlier access to acute care interventions, either in the ICU or via METs, may improve outcomes [4–7]. In addition, it has been demonstrated that METs play a role in reducing the rate of in-hospital cardiac arrest and in decreasing overall mortality [8, 9]. In healthcare systems where financial resources and intensivist availability are limited, the establishment of medical emergency teams has proven challenging. In order to conserve resources, the creation of rapid response systems that rely on a patient's usual care providers has been attempted. Recent data from Howell et al. has demonstrated that a primary team-based rapid response system was independently associated with reduced mortality [10]; however, this team did include trained physician assistants in addition to the usual medical and nursing staff.

We have previously shown that there is a significant delay between patient deterioration and ICU consultation for critically ill patients on our medical wards [11], and this delay in treatment initiation was found to be associated with increased patient mortality. Due to limited financial and human resources, we could not implement a formal medical emergency team in our institution. As a result, we implemented a low-cost, ward-based rapid response system on our medical clinical teaching unit (CTU) consisting of ward nurses and medical residents, with the senior resident acting as the intervention leader. The goal of this prospective before/after study was to examine the impact of the implementation of this ward-based team on time to intervention, code blue rates, and ICU transfer rates. In addition, we examined nursing satisfaction with the project, overall mortality rate on the CTU, and 30-day mortality rate for patients admitted to the ICU.

## 2. Materials and Methods

### 2.1. Overview of Hospital CTU Structure

Our study was conducted at a university-affiliated 350-bed acute care teaching hospital in Montreal, Quebec. The hospital has a 22-bed ICU and a 10-bed cardiac care unit (CCU). There are no step-down or high-care units within the hospital.

The CTU is a 48-bed unit with a nursing ratio of 5 : 1 during the day (7:00 h–23:00 h) and 7 : 1 during nights (23:00 h–7:00 h) which receives 70 patients per month with a median length of stay of 6 days. The CTU is not capable of providing vasoactive agents, noninvasive ventilation, or IV insulin; though resuscitative measures are commenced on the CTU in “code blue” scenarios, patients must ultimately be transferred to the ICU or the CCU in order to receive the aforementioned therapeutic interventions.

The medical team responsible for patient care on our CTU is comprised of 1 attending staff, 4 senior residents (third-year and second-year trainees), 6 junior residents (first-year trainees), and 4 third-year medical students. Trainees would have completed a minimum of eighteen weeks of acute care medicine (ICU, CCU, and emergency medicine rotations) before assuming a senior resident role. At the end of their eighteen weeks of acute care medicine, trainees are expected to recognize the unstable patient, to initiate resuscitative measures (including fluids and vasoactive agents), to identify indications for advanced ventilatory aids (noninvasive and invasive ventilation), and to be comfortable with the intubation of the airway and central venous catheterization.

CTU physician manpower availability varies depending on day of the week and time of day. On weekend days and statutory holidays, the medical staff is decreased to 1 attending staff, 2 senior residents, and 2 junior residents or medical students. On both weekday and weekend nights (20:00 h–8:00 h), there is 1 senior resident and 2 junior residents or medical students in house on call for the CTU.

### 2.2. Project Implementation and Team Education

We embarked upon a six-month educational project between January 2011 and June 2011 in order to promote awareness of the observed delays in the recognition of and response to critically ill patients on our CTU. Our initial findings were presented at both medical grand rounds and nursing rounds. Attending staff, senior and junior medical house staff, nursing directors, and bedside nurses working on the medical CTU were targeted for an educational program during the six months prior to project implementation. Rounds were conducted with small groups of bedside nurses and physicians with the following goals: (1) to explain the rationale behind the project, (2) to explain anticipated project goals, (3) to attribute the role of primary activator (afferent limb) to the nursing staff, (4) to attribute the role of primary responder (efferent limb) to the senior residents, (5) to review the criteria for system activation, (6) to explore methods of call documentation, and (7) to answer questions and to address potential concerns surrounding system activation. Posters listing system activation criteria and contact numbers were designed and placed in both nursing and physician work areas (Appendix A). Follow-up meetings with the nursing staff were arranged at the 1-, 3-, and 6-month marks after project initiation to obtain additional feedback. At the 6-month mark, a survey was distributed to the nursing staff to ensure that there was adequate buy-in and education. Additional goals of survey distribution were to obtain information concerning ease of system activation, to identify potential pitfalls in nursing staff-resident communication, and to better assess overall receptiveness of the project. The program was implemented July 1, 2011, to allow for incoming residents to be directly immersed in the project at the onset of their training.

### 2.3. Definition of Afferent and Efferent Limbs

The afferent limb of our response system, or activation component, was comprised of the CTU bedside nursing staff. As our nurses have already been trained to call for medical assistance when patient concerns arise, we elaborated a set of seven clearly ascribed calling criteria to improve early recognition of deteriorating patients.

**Figure 1:**
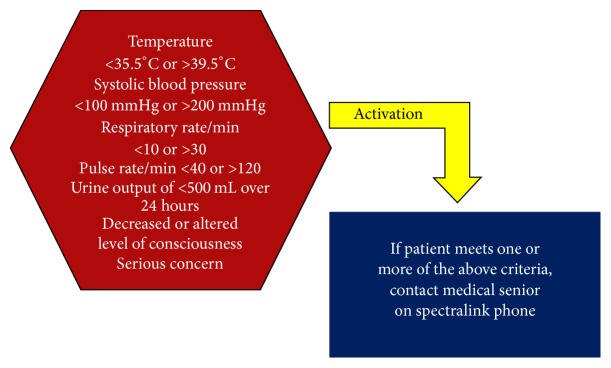
System activation criteria.

Criteria utilized for system activation included the following:

1. Temperature < 35.5°C or > 39.5°C.
2. Systolic blood pressure < 100 mmHg or >200 mmHg.
3. Respiratory rate/min < 10 or >30.
4. Pulse rate/min < 40 or >120.
5. Urine output of < 500 mL over 24 hours.
6. Decreased or altered level of consciousness.
7. Serious concern (see Figure 1).

**Figure 2:**
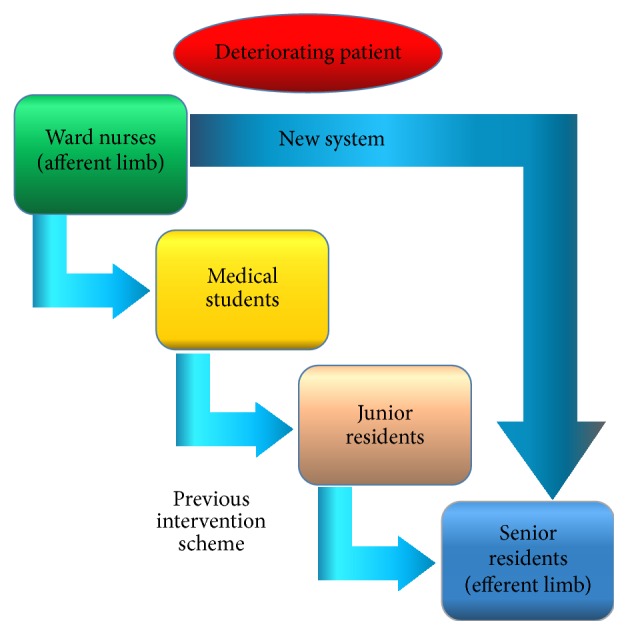
Afferent and efferent limb definition.

The efferent limb, or response component, was comprised of the CTU senior medical resident. The senior resident was contacted by the nursing staff directly via a portable phone when a patient met any one of the aforementioned system activation criteria. The senior thus served as the first-responder for patient evaluation and resuscitation. The senior, in turn, had the capability to call for medical team assistance and critical care support when deemed necessary (see Figure 2). This method of direct communication between the bedside nurse and senior resident constituted a change from our previous system, whereby the junior house staff directly caring for the patient in question has served as the first-line contact for addressing nursing concerns.

### 2.4. Study Initiation, Data Collection, and Endpoints

Our system was instituted on July 1, 2011, with data collected and reviewed until June 30, 2012. Outcomes were compared to data prospectively collected prior to initiation of the educational project (January 2010–December 2010). Our major endpoints included time from decompensation to first intervention, ICU transfer rates, and rate of code blue calls. Code blue rates on the CTU were compared with hospital-wide (excluding medical CTUs) code blue rates to account for any secular trends. The time to intervention was defined as the time from which the nursing staff noted vital signs meeting system activation criteria to the time the senior resident assessed the patient in question and subsequently (1) prescribed a treatment or (2) ordered urgent investigations or (3) called for a consultation from critical care services or (4) called a code blue.

A system activation call record spreadsheet was designed for nurses to enter patient name, hospital number, date and time of call, and reason for system activation. A similar spreadsheet was designed for senior residents to track the aforementioned data to improve accuracy. Additional data recorded by seniors included age and gender of the patient for which the call was placed, the patient's admission diagnosis, the cause or working hypothesis of his or her deterioration, poor prognostic features, time of first intervention, time of consult to critical care services (if applicable), and time of transfer to a critical care unit (if applicable). Poor prognostic factors were defined as advanced dementia and/or being a resident of a nursing home or long-term care facility, advanced cardiac and/or respiratory disease (NYHA III or IV congestive heart failure, nonrevascularizable coronary artery disease, or chronic obstructive pulmonary disease of MRC Dyspnea scale score 4 or 5), or active malignancy.

The study group reviewed all entries on a weekly basis and noted the time from patient decompensation to the time of symptom recognition and system activation. In addition, the study group reviewed all admissions to the hospital's critical care units (ICU and CCU) from the medical wards on a weekly basis between July 1, 2011, and June 30, 2012, to discern if all transfers were preceded by system activation.

Precise activation times were confirmed via retrospective chart review. Afferent limb activation times were confirmed through verification of nursing notes and by cross-checking vital sign records in our computerized patient database. Times of intervention were confirmed through revision of senior call notes and through cross-checking of dates and times of prescriptions and/or orders, investigations, or consultations. When several therapeutic measures were initiated concurrently, the earliest documented time (prescription, note, or consultation) was considered the time of therapy initiation. Additionally, times to critical care admission were confirmed via critical care service admission notes, completed by both nursing staff and ICU residents on call. When time of critical care service admission notes were unavailable, times of critical care admissions were verified using electronic records. Code blue rates, mortality rates, and ICU transfer rates were obtained through hospital databases.

Furthermore, we examined overall nursing satisfaction with the project. Upon reaching the 6-month mark, surveys were distributed to the nursing staff to better elucidate the benefits and pitfalls of the project (see Appendix B). After discussion with nursing teaching coordinators, it was decided that waiting six months prior to survey distribution would allow for adequate exposure of the bedside nurses to the activation system, thereby increasing the number of nurses having directly placed calls and thus increasing the power of the results generated.

### 2.5. Statistical Analyses

All significance testing was two-tailed and significance level was set at 0.05. Means with standard deviations and medians with interquartile ranges were used where appropriate. ICU admission rates and code blue rates were compared using incident rate ratios and Poisson regression. All statistical analyses were performed using STATA (v13).

## 3. Results

### 3.1. Patient Demographics

Activation of the new ward-based system occurred for 82 patients. The mean age of patients for which calls were placed was 70.4 years (SD: 15.6). There was a slight male predominance, with 57% of patients being male (*n* = 47).

The admission diagnoses of the patients for which calls were placed were varied, with the majority of patients admitted for respiratory pathology (*n* = 27, 33%), including pneumonia (both community acquired pneumonia and postobstructive pneumonia (*n* = 14)), chronic obstructive pulmonary disease exacerbation (*n* = 3), pulmonary embolism (*n* = 3), pleural effusion (*n* = 2), empyema (*n* = 2), acute respiratory failure (*n* = 1), hemothorax (*n* = 1), and lung mass NYD (*n* = 1). The admission diagnoses for 21% (*n* = 16) of patients for which calls were placed were malignancy-related (*n* = 7 hospitalizations related to complications of Stage IV disease), 12% (*n* = 10) were infectious disease-related (including sepsis (*n* = 4), infective endocarditis (*n* = 2), urinary tract infection (*n* = 1), diarrhea in context of previously diagnosed HIV (*n* = 1), cholangitis (*n* = 1), and cellulitis (*n* = 1)), 12% (*n* = 10) were cardiac-related (including congestive heart failure/pulmonary edema (*n* = 7), hypertensive urgency (*n* = 1) and postcardiogenic shock/cardiac arrest (*n* = 2)), and 9% (*n* = 8) were gastrointestinal disease-related (including cirrhosis (*n* = 4), gastrointestinal bleed (*n* = 3), and Crohn's flare (*n* = 1)). An additional 13% (*n* = 10) of patients for which calls were placed were admitted for other causes, including renal disease-related causes (*n* = 2, including acute kidney injury (*n* = 1) and chronic kidney disease (*n* = 1)), neurologic disease-related causes (*n* = 3, including myasthenia gravis exacerbation (*n* = 1) and delirium (*n* = 2)), rheumatological disease-related causes (*n* = 3, including vasculitis (*n* = 2) and anti-phospholipid antibody syndrome (*n* = 1)), endocrinological causes (*n* = 1, hyperglycemia), or causes unknown on account of missing medical admission records (*n* = 2) (see Table 1).

### 3.2. Call Characteristics

A total of 95 calls were placed for 82 patients during the study period, averaging 1.8 calls per week. Thirty-eight calls were placed during regular day-time working hours (defined from 8:00 h to 18:00 h), and 57 calls were placed at night (defined from 18:01 h to 7:59 h). Data surrounding precise time of system activation was missing for 7 patients. The majority of calls were placed for serious concern (24%, *n* = 23), followed by changes in mental status (21%, *n* = 20), blood pressure changes (systolic BP < 100 or >200 mmHg) (18%, *n* = 17), respiratory rate < 10 or >30 breaths/min (18%, *n* = 17), heart rate < 40 or >120 bpm (7%, *n* = 7), or multiple causes/unknown (12%, *n* = 11) (see Table 1).

### 3.3. Time to Intervention from Compensation and Critical Care Consultation

The median time from decompensation to initial intervention or ICU consultation was 5 min (IQR 1–10) during the implementation period, compared to 3.4 hours (IQR 0.6–12.4) before project implementation (*p* < 0.001). The initial intervention consisted of consultation of critical care specialties (ICU or CCU) for 34% of calls placed (*n* = 32), initiation of pharmacotherapy or oxygen therapy for 42% of calls (*n* = 40), request for STAT imaging for 4% of calls placed (*n* = 4), or execution of clinical procedures such as intubation, central venous catheterization, or arterial blood gas sampling for 12% of calls (*n* = 11). Information surrounding initial intervention was unavailable for 8 of the 95 calls (8%) due to incomplete medical records. Critical care consultations were ultimately requested after 75 of 95 calls (79%), which amounted to ICU/CCU consultation requests for 66 of the 82 patients studied (80%). Information surrounding critical care consultation was unavailable for 2 of the 82 patients on account of unavailable medical records (see Table 1).

### 3.4. ICU Admissions and APACHE II Scores

We observed a reduction in the number of ICU admissions from the CTU, with an admission rate of 4.8/1000 patient days (±2.2) during the control period, reduced to 3.3/1000 patient days (±1.4) during the intervention period (IRR: 0.82, *p* = 0.04 (CI 95%: 0.69–0.99)). The average APACHE II scores of patients admitted to the ICU decreased, with an APACHE II score of 25.2 (CI 95%: 23.0–27.5) after intervention, compared with 28.4 (CI 95%: 26.3–30.5) during the control period (*p* = 0.04) (see Table 1).

### 3.5. Code Blue Rates

Code Blue rates on the CTU fell, with a decrease from 2.2/1000 patient days (±1.6) during the control period to 1.2/1000 patient days (±1.3) during the intervention period (IRR: 0.51, *p* = 0.02 (CI 95%: 0.30–0.89)). Comparatively, there was no change in code blue rates throughout the rest of the hospital during the same period, with a hospital-wide code blue rate during the control period of 1.2/1000 patient days (±0.53) and the code blue rate during the intervention period of 1.1/1000 patient days (±0.57) (IRR: 0.93, *p* = 0.56 (CI 95%: 0.72–1.20)) (see Table 1).

### 3.6. Mortality Data

Mortality rates for patients admitted to the CTU did not change as a result of our intervention. During the control period, mortality was 10.1% (±4.3%), compared to 10.9% (±3.7%) during the intervention period (*p* = 0.64). The 30-day ICU mortality rate also did not change, with a mortality rate during the intervention period of 34.5% (21.9–47.1) compared to 29.3% (18.8–39.9) during the control period (*p* = 0.53) (see Table 1).

### 3.7. Poor Prognostic Factors

Of the 82 patients for which calls were placed, a total of 52 were identified as having poor prognostic factors, including advanced dementia or being a permanent resident of a nursing home or of a long-term care facility (9%, *n* = 8), suffering from advanced cardiac and/or respiratory disease (27%, *n* = 22), or having an active malignancy at the time of admission (27%, *n* = 22) (see Table 1).

### 3.8. Changes in Level of Intervention

Of the 82 patients for which calls were placed, a total of 72 had requested Level of Care (LOC) 1, indicating provision of all potential life-saving measures and transfer to a monitored care unit (ICU or CCU). After system activation, a total of 8 patients (11%) underwent changes in their level of intervention, with change from LOC 1 to LOC 3 and decision to continue care without transfer to a monitored setting.

### 3.9. Nursing Satisfaction

In total, 20 members of the nursing team (*n* = 34) were surveyed at random at the project's six-month mark to better conceptualize the ease of system activation, comfort of nurse-resident communication, and perception of project effectiveness. Surveys were conducted anonymously to facilitate nonbiased feedback. The survey was comprised of a total of 9 questions (7 YES/NO questions, 1 multiple choice question, and 1 open feedback area for additional questions, comments, and concerns) (see Appendix B).

Of the nurses surveyed, 70% (*n* = 14) reported having activated the new ward-based system or having directly alerted the senior resident (as opposed to the junior resident) about a deteriorating patient. Among the 14 nurses having used the new ward-based system, 88% (*n* = 12) reported that the senior resident responded in a timely manner, 88% (*n* = 12) believed that the senior responding to their call was courteous and respectful, and 93% (*n* = 13) felt comfortable with the activation criteria, with 79% (*n* = 11) reporting that the system calling criteria was clear to them. Interestingly, 6 out of 14 nurses expressed that they encountered difficultly knowing when to activate the system on occasion, primarily due to the proximity and accessibility of junior house staff and to occasional difficulties contacting the senior (busy phone lines or no response). Overall, however, 93% of the nurses surveyed (*n* = 13) supported the project, agreeing that our system ultimately resulted in better patient outcomes.

## 4. Discussion

Our study demonstrated that a ward-based response system consisting of the bedside nurse acting as the afferent limb and the senior resident as the efferent limb can decrease code blue rates and ICU transfer rates. The time to intervention after deterioration was also shortened. In contrast, we did not demonstrate a decrease in overall mortality or 30-day mortality after ICU transfer.

The ideal composition of rapid response teams remains unknown [12]. Though the advantages of an intensivist-led MET are intuitive, resource limitations in many institutions render the creation of teams led by senior physicians trained in acute resuscitation economically and structurally unfeasible. Interestingly, a recent retrospective study by Morris et al. suggests that resident-led rapid response systems may have similar outcomes to attending intensivist-led events [13]. Additionally, the work of Rothberg et al. demonstrates that a hospitalist-led MET can succeed in decreasing code call rates; however, a reduction in overall mortality has yet to be observed [14]. A recent system designed by Howell et al., whereby the MET was comprised of the patient's usual care providers, suggests that primary team-based implementation of a rapid response system can be independently associated with reduced unexpected mortality and may offer a more cost-effective approach to designing and implementing rapid response teams [10].

In contrast to Howell et al.'s system, in which the MET was comprised of the patient's primary nurse, the floor's senior nurse (nurse educator or specialty), the patient's primary house officer or licensed independent practitioner, and a respiratory therapist in the case of respiratory criteria activation, our local ward-based system was substantially smaller, comprising only the patient's primary bedside nurse (afferent limb) and the CTU senior resident (efferent limb). Nonetheless, our study demonstrated that this system consisting of the bedside nurse acting as the afferent limb and the senior resident as the efferent limb can decrease code blue rates and ICU transfer rates. The time to intervention after deterioration was also shortened.

Despite the decrease in APACHE II score from 28.4 to 25.2 among patients admitted to ICU after system implementation, which would suggest a potential decrease in the predicted risk of death from approximately 64% to 55%, we did not demonstrate a decrease in overall mortality or 30-day mortality after ICU transfer. Although some studies have shown a decrease in 30-day ICU mortality [15], we did not demonstrate this with our intervention. It is possible that the number of ICU transfers in total was very small. Despite our limited staffing, our results are consistent with those of a meta-analysis of traditional rapid response teams, which found an overall 34% reduction in out-of-ICU cardiac arrests (OR 0.66, 95% CI 0.54–0.80) [16].

Overall mortality rates on the CTU did not decrease with the implementation of the local response system. Rapid response teams have not consistently shown a decrease in overall mortality [17–19]. It is also quite possible that interventions such as METs would not change mortality rates appreciably as most deaths are not preventable and a much larger sample size would have been required to demonstrate a difference [16].

Our results are relevant to both clinicians and administrators. The structure of our system allows for care-giver continuity and preserves educational opportunities by allowing residents to directly participate in the management of critically ill patients. Our system requires no additional clinical staffing and thus no additional funding, which could prove extremely advantageous in schemes where both human and economic resources for rapid response team creation are limited. In addition, our system's activation does not automatically disrupt the day-to-day activities of nursing, respiratory therapy, and intensive care workers. Furthermore, not all hospitals have access to trained nurse practitioners.

Our study reflects the trend toward rapid response teams implementing DNRs and modifying levels of intervention. In our study, levels of care were changed for 11% of the patients (*n* = 8) for which calls were placed. Of these eight patients, all had one or more identified poor prognostic factor. A similar trend with rapid response teams modifying care goals was observed by Chen et al. [20], where such teams did in fact result in an increase in DNR orders compared to hospitals without rapid response systems, although the magnitude of this effect was small (approximately 4 additional DNR orders per 10,000 admissions).

Additionally, the role of the MET in end-of-life care was recently explored in work by Jones et al. [21]. The capacity of our efferent limb to modify care goals at a rate higher than previously observed could be a representation of case familiarity since the structure of our response system preserves continuity of patient care.

The major limitation of our study is that it is a single-center study which involves senior residents who have had adequate critical care exposure. Nonetheless, the level of critical care exposure of our senior internal medicine residents reflects that of their colleagues trained in internal medicine at other Canadian university-affiliated institutions. Prior to the implementation of a similar ward-based response system at other centers, one would need to consider the level of critical care training of the efferent limb, as well as the acute care exposure and competence of the nursing staff, the availability and willingness of the health care team to embark upon a similar project, and the ease of access to the critical care environment.

## 5. Conclusion

Our local ward-based response system succeeded in achieving a significant reduction from the time of patient decompensation to initial intervention without necessitating additional usage of financial or human resources. Our system achieved a reduction in code blue rates on our CTU and led to an overall decrease in the number of transfers of CTU patients to the ICU. Our system is novel in that it is centered on a ward resident-based efferent limb, it requires no additional human or economic resources for implementation, and it lends itself to continuity of patient care. Expansion of our system to other centers is required to further appreciate the magnitude and generalizability of these results.

## Figures and Tables

**Figure 3:**
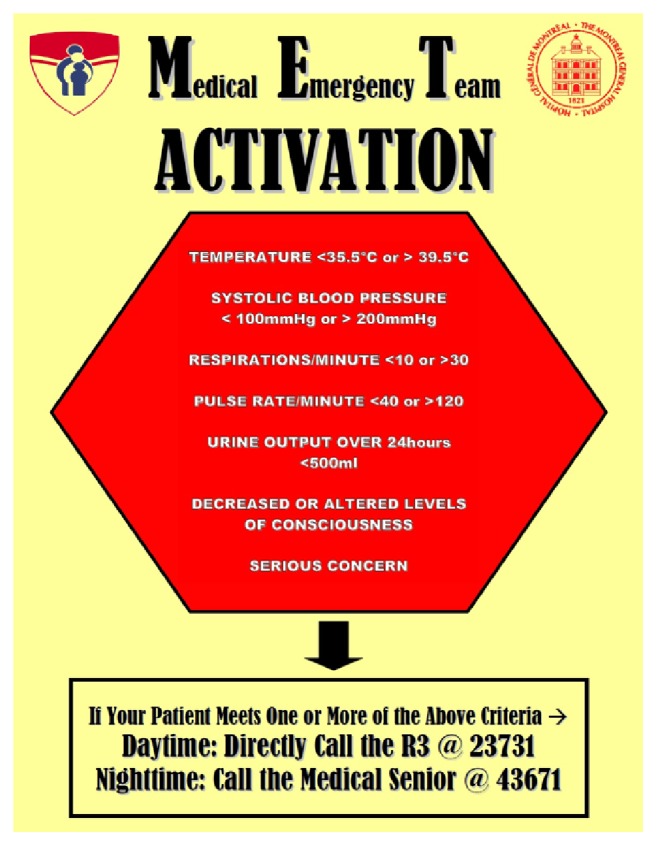
Sample poster: system activation criteria.

**Figure 4:**
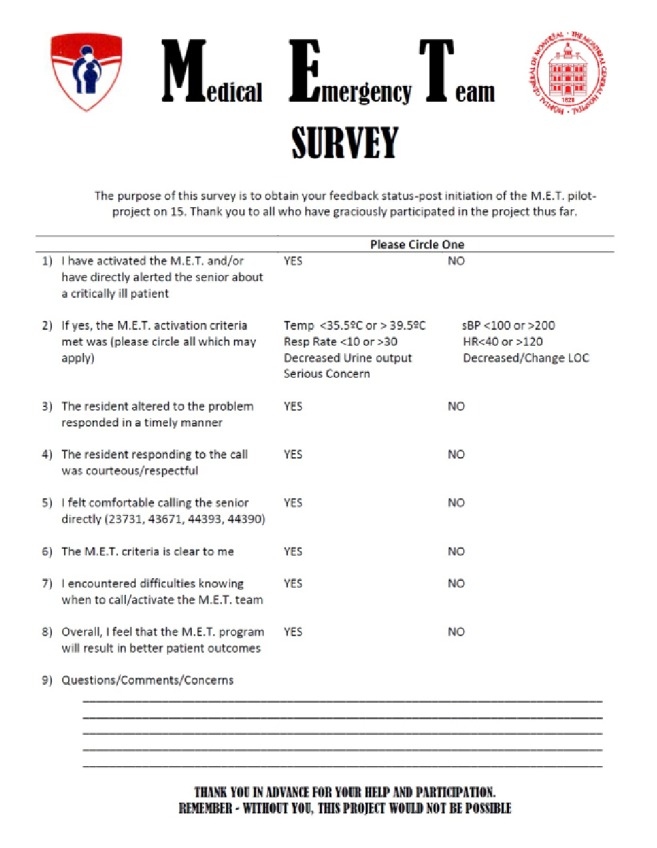
Sample of nursing survey.

**(a):** 

*n*	82
Male (%)	47 (57)
Age (years), mean (SD)	70.4 (15.6)
Admission diagnosis by category	
(i) Respiratory (%)	27 (33)
(ii) Malignancy (%)	16 (21)
(iii) Infectious disease (%)	10 (12)
(iv) Cardiac (%)	10 (12)
(v) Gastrointestinal (%)	8 (9)
(vi) Other (%)	11 (13)

**(b):** 

Total calls (mean per week)	95 (1.8)
(i) Daytime calls (8:00 h–18:00 h) (%)	38 (40)

Activation triggers, call characteristics	
(i) Blood pressure (%)	17 (18)
(ii) Heart rate (%)	7 (7)
(iii) Respiratory rate (%)	17 (18)
(iv) Change in mental status (%)	20 (21)
(v) Serious concern (%)	23 (24)
(vi) Multiple or unknown (%)	11 (12)

Initial intervention after system activation	
(i) Critical care consultation (%)	32 (44)
(ii) Initiation of pharmacotherapy oxygen therapy (%)	40 (42)
(iii) STAT imaging (%)	4 (4)
(iv) Clinical procedures (intubation, central line, and ABG) (%)	11 (12)
(v) Information not available	8 (8)

Patients with poor prognostic factors (*n*)	52
(i) Advanced dementia or long-term care facility (% total)	8 (9)
(ii) Advanced cardiac or respiratory disease (% total)	22 (27)
(iii) Active malignancy (% total)	22 (27)

**(c):** 

	Before intervention	After intervention	*p* value
Time from decompensation to intervention (min) (IQR)	204 (1–10)	5 (0.6–12.4)	<0.001
ICU admissions (per 1000 patient days)	4.8	3.3	0.04
APACE II scores (mean) (CI 95%)	28.4 (26.3–30.5)	25.2 (23.0–27.5)	0.04
CTU code blue rates (per 1000 patient days)	2.2	1.2	0.02
Hospital-wide code blue rates (per 1000 patient days)	1.2	1.1	0.56
CTU mortality (%)	10.1	10.9	0.64
30-day ICU mortality (%)	29.3	34.5	0.53

## Appendix

### A. Sample Poster: System Activation Criteria

See Figure 3.

### B. Sample of Nursing Survey

See Figure 4.
